# Measuring Surface and Interfacial Tension In Situ in Microdripping Mode for Electrohydrodynamic Applications

**DOI:** 10.3390/mi11070687

**Published:** 2020-07-16

**Authors:** Karim I. Budhwani, Gerald M. Pekmezi, Mohamed M. Selim

**Affiliations:** 1 CerFlux, Inc., Birmingham, AL 35205, USA; 2 School of Medicine and School of Engineering, University of Alabama at Birmingham (UAB), Birmingham, AL 35294, USA; gPekmezi@uab.edu (G.M.P.); mSelim@uab.edu (M.M.S.)

**Keywords:** nanofabrication, tissue engineering, microphysiological systems, drug delivery systems, electrospinning, electrospraying, surface tension, computational fluid dynamics

## Abstract

Walking on water is made possible, at least for tiny insects, by molecular interaction at the interfaces of dissimilar materials. Impact of these interactions—surface tension (SFT) and, more broadly, interfacial tension (IFT)—is particularly evident at micro and nano sizescales. Thus, implications of walking on water can be significant for SFT or IFT (S/IFT)-driven nanofabrication technologies, such as electrohydrodynamic atomization (EHDA), in developing next generation biomimetic microphysiological systems (MPS) and drug delivery systems (DDS). However, current methods for estimating S/IFT, based on sessile drops or new surface formation on a ring or plate, are unsuitable for integration with EHDA assemblies used in electrospinning and electrospraying. Here, we show an *in situ* method for estimating S/IFT specifically devised for EHDA applications using signal processing algorithms that correlate the frequency and periodicity of liquid dispensed in EHDA microdripping mode with numerical solutions from computational fluid dynamics (CFD). Estimated S/IFT was generally in agreement with published ranges for water–air, 70% ethanol–air, chloroform–air, and chloroform–water. SFT for solutions with surfactants decreased with increasing concentrations of surfactant, but at relatively higher than published values. This was anticipated, considering that established methods measure SFT at boundaries with asymmetrically high concentrations of surfactants which lower SFT.

## 1. Introduction

With one in two men and one in three women poised to be diagnosed with cancer [1], disease modeling with biomimetic microphysiological systems (MPS) and targeted drug delivery systems (DDS) is of keen interest in micro and nanoscale processing. EHDA fabrication technologies, such as electrospinning (ESp) and electrospraying (ESy), offer promising pathways in both MPS and DDS applications. ESp is well established and continues to build momentum in tissue engineering but is eclipsed by soft lithography in MPS for investigating disease progression and treatment evaluation. However, the porosity of scaffolds, which is integral to *in vivo* barrier and interface functions, is either entirely absent in MPS, like lab-chip systems, or introduces considerable cost, complexity, and an unrealistic uniformity in pore geometry. Nanofibrous porous scaffolds produced using ESp would be more suitable for recapitulating the *in vivo* tissue microenvironment in MPS, particularly for modeling organ–capillary transport, the air–liquid interface, and tumor progression [2]. Mechanisms to improve the throughput of nanofibrous scaffolds continue to gain momentum [3]. Similarly, ESy can be used to manufacture micro and nanocontainers [4,5,6] that reduce systemic toxicity and increase efficacy by delivering therapeutics only to the target site. While emulsification is the established method for manufacturing gas-filled microbubbles, used clinically as ultrasound contrast agents [7] (UCA), ESy offers considerable advantages for fabricating drug-loaded core-shell devices; specifically, in terms of higher encapsulation efficiency, near monodisperse distribution, narrow size distribution [8,9], and versatile morphology options including core-shell nanofibers [10,11]. Combining the two ideas, EHDA can also be used to produce bioactive nanofibrous scaffolds for tissue engineering applications that encapsulate growth factors or genetic material and are capable of programmed delivery of these to engineered microtissue over time [12,13]. Aside from tissue engineering and biomedical applications, EHDA is integral to a wide range of technologies, including filtration, renewable and green energy, and electrospray thrusters for space propulsion applications. New multiplexing and planar arrays [14], and mechanisms for optimizing these [15], continue to emerge to meet the increasing throughput demands of such applications. Other limiting factors, such as clean room fabrication, are being overcome with additive manufacturing capabilities [16].

However, the very principle governing Taylor cone formation and jet breakup in EHDA—interplay among S/IFT, gravity, mechanical forces, and electric fields—adds a dimension of instability and unpredictability [9,17] to the process. Variations in S/IFT from alterations in material composition or concentration, equipment, or environment, can dramatically alter output, efficiency, or both. Consider variations in temperature (energy of particles at the interface), humidity (saturation and concentration gradients), gravity, and other forces (centrifugal, centripetal, electromagnetic, mechanical) driving particles at material interfaces closer or further apart. Accounting for these *in situ* could assist in extemporaneous tuning of parameters to compensate for such variances. For instance, applied electric fields can be reduced in response to lower than expected S/IFT from changes in concentration of added pharmaceutical surfactants. Additionally, identification of S/IFT values asynchronously would be useful in the analysis and post-processing phase of EHDA.

**Figure 1:**
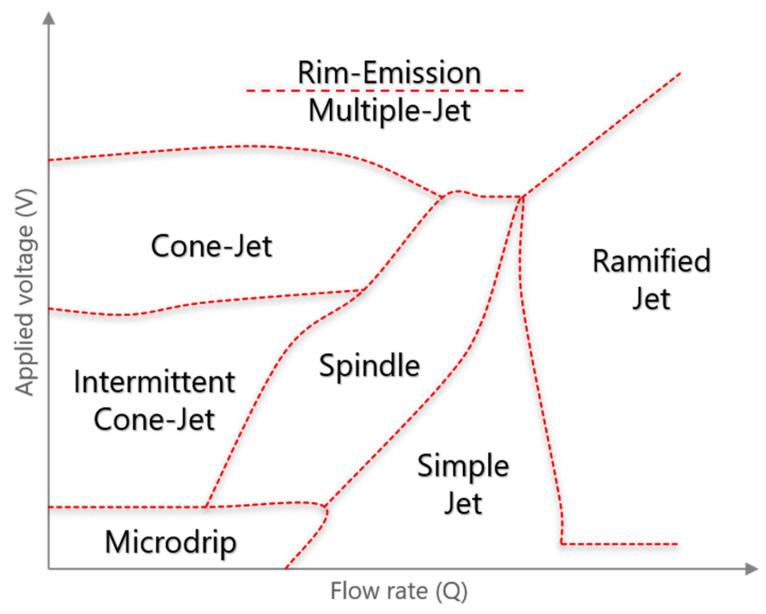
Approximate regimes of electrohydrodynamic atomization (EHDA) modes shown as a function of applied voltage (V) and flow rate (Q).

It is informative to briefly review the merits of the EHDA microdripping mode as a starting point for developing computational modeling. EHDA processing regimes as a function of applied voltage (V) and flow rate (Q) are loosely illustrated in Figure 1. The Microdripping mode is generally observed for combinations of very low flow rates (Q = Q_low_) and very low, even zero, applied voltage (V = V_min_) [18,19,20,21,22,23]. Due to the relatively low influence of mechanical and electrical forces in this mode, drops are dispensed almost solely due to the interplay between S/IFT and gravity. With electromechanical stresses muted, at V_min_ = 0, this mode is particularly suited for *in situ* measurement of S/IFT. Moreover, as one would intuitively expect, particle production frequency in this mode is relatively very low—from near zero Hz to low kHz. Another interesting consequence of the virtually nonexistent influence of V_min_ is that there is no cone formation at the tip of the nozzle. Instead, as the solution flows through the assembly to the nozzle tip, the drop at the tip continues to grow primarily as a function of SFT, capillary diameter, and Q_low_ until the weight (gravity) of the drop overcomes SFT. At this point, one part of the drop from the nozzle tip is dispensed, while the remaining solution relaxes to regain its original smaller shape at the tip of the nozzle. This mode is also characterized with near monodisperse particle sizes.

**Figure 2:**
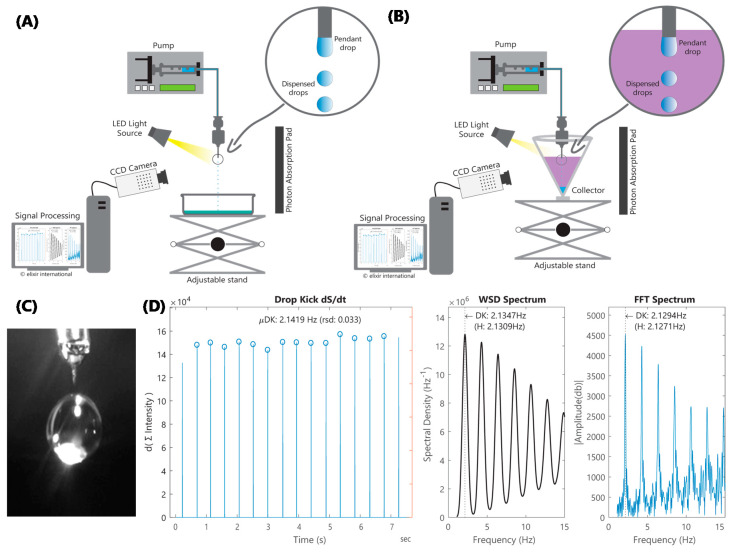
Schematic illustration of the modified EHDA setup for *in situ* (**A**) surface tension (SFT) and (**B**) interfacial tension (IFT) measurements. In both cases, a light-emitting diode (LED) light source is pointed slightly below the tip of the EHDA nozzle, while a charge-coupled device (CCD) camera captures images at a minimum 200 frames per second (fps). (**C**) An image of a dispensed drop captured by the camera. (**D**) Three panels showing the signal derived from captured image sequence (video) in the time domain, where dS/dt is the derivative of signal intensity with respect to time, which highlights peak intensities at drop locations, Welch’s power spectral density (WSD) and fast Fourier-transform (FFT) in the frequency domain. Both WSD and FFT indicate that the signal is not only periodic, given that almost all the power is in the fundamental frequency and its multiples (harmonics), but that the fundamental frequency is 2.13 Hz, which is close to the average drop rate.

Many refinements and alternatives have been developed for Worthington’s classic Pendant Drop [24], from reducing measurement times to accounting for dynamic elements [25,26,27]. However, established S/IFT measurement methods, including du Noüy ring and Wilhelmy plate, are not suited for EHDA integration. Typically, EHDA assemblies include pumps that dispense solutions at controlled rates through precision diameter nozzles, from which the solution is spun or sprayed. This setup precludes rings or plates, which are central to the du Noüy and Wilhelmy methods. Similarly, kinetics and forces involved in spinning-drop and bubble-pressure tensiometry [28] are incompatible with EHDA, where the solution is neither in a state of perturbation nor in equilibrium. On the other hand, pendant or dispensed drops in Worthington’s pendant drop, stalagmometric, and drop volume methods, seem better suited for EHDA integration. These methods derive SFT from the shape of pendant drops, average weight of collected drops, and residual drop-volume on the nozzle tip after the drop is dispensed, respectively. Bridging elements from these with statistical analysis, digital signal processing (DSP) algorithms and computational fluid dynamics (CFD) simulations [29,30], we devised a drop kinetics (or “drop-kicks”) tensiometry platform that seamlessly integrates with EHDA configuration, as shown in Figure 2A,B. Our approach, which is notably different from the du Noüy ring and Wilhelmy plate methods, offers considerable advantages including form-factor, interface characteristics, flow kinetics that are germane to EHDA and the capacity for IFT determination, which is particularly relevant for coaxial EHDA applications. Our approach also differs from pendant drop in terms of flow kinetics (dispensed vs. sessile drops) and computation (periodicity and frequency vs. radii of surface curvature). The use of drop frequency measurements enables us to compute IFT under dynamic conditions, where inertial effects are not negligible and the equilibrium assumption of the Young–Laplace equation is not valid. This precludes the use of the Pendant Drop method, which is typically considered appropriate for drop frequencies up to 1 Hz [31].

## 2. Materials and Methods

EHDA setup modifications include the addition of a computer-connected charge-coupled device (CCD) camera, a light source, and a dark mat to absorb scattered light. For IFT, the lighter fluid is placed in a conical collector, with the nozzle-tip positioned just under the meniscus, while the heavier fluid is dispensed from the nozzle. Video images are captured (Figure 2C) at 200+ frames per second (fps) as fluid is dispensed in microdripping [20] mode—low flowrate (Q) and 0 kV applied voltage (V)—to reduce variables and multiphysics complexity. Nozzle diameter and flow velocity were chosen to maximize droplet frequency, while maintaining a sufficiently low Weber number; the Weber number is the ratio of inertial force to SFT. This is to ensure that SFT has a significantly higher impact, compared to fluid viscosity, on flow kinetics. Custom MATLAB^®^ (2017a, MathWorks, Inc., Natick, MA, USA) modules were developed to derive time domain signals from the flux in the columnar sum of pixel intensities of light reflected from the dispensed drops in the captured frames. The algorithm then analyzes this signal to determine periodicity and frequency (Figure 2D).

If the signal is periodic, S/IFT is determined by cross-referencing it against periodicities and frequencies computed using the same algorithms previously run on volume-fraction output (instead of reflected light intensity) from a coupled level-set volume of fluid (VOF) model simulation.

### 2.1. Computational Model

**Figure 3:**
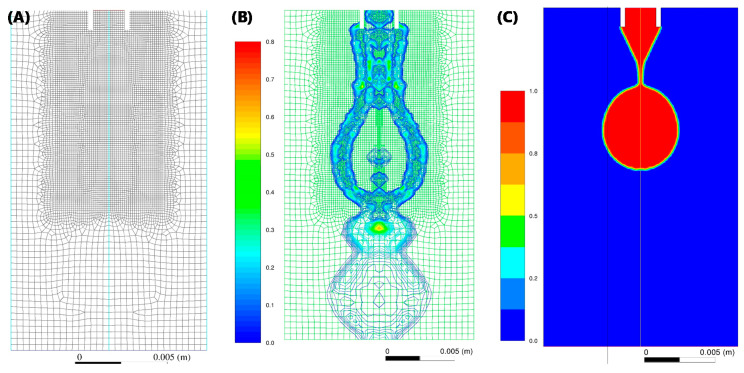
Computational fluid dynamics (CFD) domain, adaptive mesh, and drop kinetics simulation. (**A**) Domain showing tip of the nozzle and non-adaptive mesh. (**B**) Mesh adaptation function. (**C**) Volume fractions from a detaching drop in two-phase simulations of dispensed drops.

The axisymmetric computational domain consists of a 2D cross section of stainless steel 14 g needle (1.600/2.108 mm inner/outer diameter). In order to improve performance, only the needle tip was included in the domain. Further to this, it was assumed that the needle tip has a smooth inner and outer surface with perfect circular inner and outer diameters, as shown in Figure 3A, with a non-adaptive mesh. Applying levels of mesh adaptation functions (Figure 3B) had no significant impact, indicating mesh-independence.

The parameters used in the simulations are summarized in Table 1. Maximum iterations per time step were set to 10 for all simulations. Output from simulations was analyzed using custom Python modules for post-processing speed, consistency, and accuracy. Please refer to the Supplementary Material section for Ansys^®^ Fluent^®^ (18.0, Ansys, Inc., Canonsburg, PA, USA) project runtime parameters.

### 2.2. Signal Processing and Spectral Analysis

The fast frame video captured using LabView^®^ (2016, National Instruments, Austin, TX, USA) was processed using a custom MATLAB^®^ program to confirm the periodicity of dispensed droplets and calculate droplet frequencies from the input signal comprising of a series of image frames. Briefly, the algorithm comprised of transforming 2D image frames to a 1D array of columnar intensity sums as a means to rapidly pinpoint the droplet’s position and size in each frame. The input signal, which is essentially an array of 2D image frames, is thus converted into an array of 1D arrays, which forms a convenient numerical proxy for the original fast frame video captured by the LabView^®^ module. A visual representation of this array of 1D arrays is shown in the first panel in Figure 2D. Next, spectral density and period are computed by the algorithm using Welch’s method. Spectral density of a signal is simply the power of the signal at different frequencies. The algorithm starts by converting the input signal from the time to frequency domain to obtain the periodogram/spectrum. Compared to the standard periodogram spectrum and Bartlett’s method, Welch’s method reduces noise by compromising (reducing) frequency resolution. This is advantageous when the signal contains noise from imperfect or finite data and where noise reduction is desired. A visual representation of the Welch spectral density computed is shown in the second panel in Figure 2D. The algorithm also performs a confirmatory Fast Fourier-Transform (FFT) analysis. It is important to note that FFT is “conjugate symmetric” with a 2-sided spectrum function of both positive and negative frequencies (−Fs/2 to +Fs/2). The algorithm computes a 1-sided spectrum (with twice the amplitude) based on a 2-sided spectrum and signal length equal to the original signal. Absolute values of the complex-valued FFT are computed to extract the 1-sided magnitude and filter out phase data. A visual representation of the FFT is shown in the third panel in Figure 2D. Please refer to Supplementary Material section for LabView^®^ and MATLAB^®^ source files.

### 2.3. Experimental

Minor changes were made to the experimental setup described in earlier studies. Briefly, a high precision syringe pump was connected to a stainless steel 14 g needle (1.600/2.108 mm inner/outer diameter; purchased from ramé-hart instrument co.) using 1/16” chemical resistant autoclavable Tygon^®^ tubing (Saint-Gobain Corporation, La Défense, Courbevoie, France). For flow rates below 500 µL/min, a BeeHive^®^ MD-1020 (Bioanalytical Systems, Inc., Lafayette, IN, USA) syringe pump was used, while a KDS-410 (kdScientific^®^, Holliston, MA, USA) was used for flow rates ranging from 500 µL/min to 20 mL/min. A high-speed Teledyne Dalsa Genie^®^ CR-GM00-H6401 camera (300 fps, Teledyne DALSA, Waterloo, Ontario, Canada) fitted with extension tubes and a 10x macro lens and connected to a computer system running custom LabView (LabView^®^ 2016, Vision Acquisition Software^®^ 2017, and Vision Development Module^®^ 2016) and MATLAB (MathWorks^®^ MATLAB^®^ 2017a) programs, was positioned 40 mm away from the needle tip, normal to which, a focused LED light source was also positioned 40 mm away from the needle. As noted earlier, to ensure a low Weber number, parameters such as nozzle diameter, flow rate, density, and viscosity must be appropriately configured. Droplet frequencies near 1–5 Hz minimize computational resources and also control experimental variance. In addition, care must be exercised to manage conditions such as atmospheric saturation, nozzle-plane leveling, and surrounding vibration. Tubing and apparatus must be examined to ensure that no contaminants are inadvertently introduced in the system. Furthermore, pumps must be calibrated and fine-controlled to ensure smooth and accurate dispensing of solution, which in turn, ensures a near-constant flow velocity. Ethanol (70% *v*/*v*) was purchased from Ricca Chemical Company (Arlington, TX, USA). Saline (0.9% NaCl with trace HCl or NaOH for pH adjustment) was purchased from Hospira (Lake Forest, IL, USA). Molecular biology grade chloroform (with 0.75% Ethanol preservative) and non-ionic surfactant Tween^®^ 20 (Polyoxyethylene-20-sorbitan Monolaurate) were purchased from Fisher Scientific (Fair Lawn, NJ, USA). All experiments were at least performed in triplicate. At least 3 measurements were made for all experimental observations.

## 3. Results

**Figure 4:**
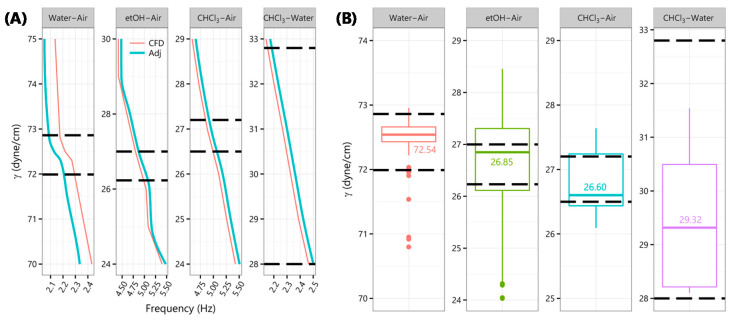
Estimating SFT and IFT. (**A**) Frequencies from CFD numerical solutions for discrete S/IFT values were interpolated, labeled “CFD,” and adjusted, “Adj”, −4% for Water–Air and +1.5% for other interfaces. Bold dashed lines indicate published [32,33] range of S/IFT for respective interfaces. (**B**) S/IFT derived by cross-referencing *in situ* recorded “drop-kicks” frequencies against corresponding “Adj” frequencies from CFD numerical solutions. Aside from a few outliers, resulting S/IFT were in agreement with published ranges, particularly for Chloroform–Water IFT. Flowrate (Q) for CHCl3–Air SFT was 3 mL/min, all others were at 5 mL/min.

Frequencies for discrete S/IFT from CFD solutions were interpolated to obtain reference values at finer S/IFT intervals (included in the Supplementary Material). The resulting Chloroform–Air SFT and Chloroform–Water IFT curves were approximate linear functions of “drop-kicks” frequency. However, 70 *v/v %* Ethanol–Air and Water–Air SFT (Figure 4A) were curvilinear. Water–air SFT sported a parabolic feature between 70 and 75 dyne/cm with a virtual flatline between 72 and 72.8 dyne/cm. The flatline was resolved by replacing it with two points at 72.5 ± 0.3 dyne/cm with simple frequency averages for either side. It was also observed that water drops were dispensed slightly faster in simulation. A straight-line adjustment (labeled “Adj” in Figure 4A) of −4% for water, and +1.5% for other cases, was applied.

Figure 4B shows S/IFT obtained by cross-referencing *in situ* recorded “drop-kicks” frequencies with those from CFD solutions along with corresponding published values [32,33] (dashed lines).

**Figure 5:**
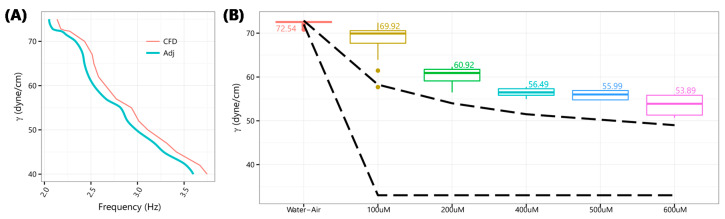
Effect of surfactants and additives on SFT. (**A**) Frequencies from CFD numerical solutions for a wider range of discrete Water–Air SFT values interpolated, labeled “CFD,” and adjusted, “Adj,” −4%. (**B**) “Drop-kicks” frequencies cross-referenced against Water–Air “Adj” frequencies to derive SFT for Water–Air and various micromolar concentrations of aqueous Tween^®^ 20 solutions. Bold dashed lines indicate published ranges [34,35,36] of SFT. SFT decreased with increasing concentrations of Tween^®^ 20 but at relatively higher than published values. This was anticipated considering that established methods measure SFT at boundaries with asymmetrically high concentrations of surfactants which lower SFT.

Finally, for solutions with surface-active additives or surfactants, which are widely used in pharmaceutical applications, we expected our SFT estimates to be higher. This is because it is energetically favorable for surfactants to orientate toward the surface, which in turn, lowers the SFT at the interface. The plate/ring setup, thus, makes it so that SFT is measured at boundaries with asymmetrically high concentrations of surfactants. As shown in Figure 5, we reported a reduction in SFT with increasing concentrations of Tween^®^ 20 aqueous solutions similar to published profiles but, as anticipated, at relatively higher values of SFT [34,35,36].

## 4. Discussion

The level-set method [37] is an interface-tracking method for computing two-phase flows with topologically complex interfaces, wherein the interface is captured and tracked by the level-set function. Because this function is smooth and continuous, its spatial gradients can be accurately calculated, which in turn, produces accurate estimates of interface curvature and SFT caused by the curvature. However, this method is found to be deficient in volume conservation [38]. On the other hand, VOF applied to a fixed Eulerian mesh [39] is naturally volume-conserving, as it computes and tracks volume-fraction of a particular phase in each cell rather than the interface itself. In this model, a single set of momentum equations is shared by the fluids, and the volume-fraction of each fluid in each computational cell is tracked throughout the domain by solving a continuity equation for the volume-fraction of one or more phases. The weakness of the VOF method lies in calculating spatial derivatives, since the VOF function (volume fraction of a particular phase) is discontinuous across the interface. To overcome the deficiencies of both, a coupled level-set VOF [40] approach was adopted. Setting interface Γ as zero level-set, the level-set function *φ* is defined as a signed distance to interface *φ* {*x,t*} and can be expressed as:

(1) $$\varphi \left\{x,t\right\}=\{\begin{array}{cc} +\left|d\right| & \mathrm{if}\;x\in \mathrm{the primary phase}\\ 0 & \mathrm{if}\;x\in \Gamma \\ -\left|d\right| & \mathrm{if}\;x\in \mathrm{the secondary phase} \end{array}$$

where *d* is distance from the interface. Similarly, evolution of the level-set function is:

(2) $$\frac{\partial }{\partial t}\left(\varphi \right)+\nabla \cdot \left(\varphi \vec{v}\right)=0$$

where $\vec{v}$ is the flow velocity.

Since SFT acts to minimize surface area of the interface, it gives rise to effects such as pressure discontinuity at the interface and capillary effects at adhesive walls. The continuum surface force (CSF) model [41,42] results in a source-term in the momentum equation from the addition of SFT to the VOF calculation.

(3) $$\frac{\partial }{\partial t}\left(\rho \vec{v}\right)+\nabla \cdot \left(\rho \vec{v}\vec{v}\right)=-\nabla p+\nabla \cdot \tau +\rho \vec{g}-{\vec{F}}_{sf}$$

where ${\vec{F}}_{sf}$ is the force from SFT effects, *ρ* is volume-averaged-density, p is the pressure, and τ is the deviatoric stress tensor. For two phases, the force from SFT effects becomes:

(4) $${\vec{F}}_{sf}=\sigma_{ij}\frac{\rho k_{i}\nabla \alpha_{i}}{\frac{1}{2}\left(\rho_{i}+\rho_{j}\right)}$$

where $\sigma_{ij}$ is the coefficient of SFT between primary phase *i* and phase *j*, *k_i_* is local mean interface curvature for *i*, and $\alpha_{i}$ is volume-fraction of *i*. Equation (4) shows that SFT source-term for a cell is proportional to the average density in the cell. For two-phase systems, volume-fraction-averaged density becomes:

(5) $$\rho =a_{j}\rho_{j}+\left(1-a_{j}\right)\rho_{i}$$

Although, it is established that temperature plays an important role in S/IFT, temperature was explicitly measured and controlled on the experimental side. Given the temperature ranges expected in a controlled laboratory setting, on the computational side, temperature was an implicit parameter with negligible effect on viscosity and density of fluid. Numerical solutions were obtained for these governing equations over the domain shown in Figure 3A. Applying levels of mesh adaptation functions (Figure 3B) had no significant impact (Figure 3C), indicating mesh-independence. For preliminary evaluation of this approach and its applicability to a broad range of fluids, simulations and tests were run for: (i) Chloroform (CHCl_3_), an organic solvent with high density but low dynamic viscosity; (ii) 70 *v*/*v %* ethanol (etOH), with low density but high viscosity; (iii) water.

The “drop-kicks” frequency and periodicity of signals, derived from both CFD models and CCD videos from experiments, were determined by first computing an average drop rate. This was used as bracketing input for DSP algorithms, including Welch’s spectral density (WSD), to determine the fundamental frequency, harmonics, and power at those frequencies (Figure 2D). Periodicity is an important indicator of the requisite uniformity of volume dispensed in each drop. Solutions with aperiodic (no uniformity) or quasiperiodic signals, where uniform volume is dispensed over a series of drops, are excluded from the scope of this work. Runtime parameters and scripts encoding algorithms for analyzing signals from both CFD and CCD are included under the Supplementary Material.

## 5. Conclusions and Outlook

Tissue engineering applications such as MPS—for modeling disease progression and evaluating efficacies of new treatments—can be enhanced by replacing soft lithography-fabricated membranes with biomimetic nanofibrous scaffolds produced using ESp. With improved configuration control of ESp, MPS parameters could be refined to enable control and capabilities which are simply not possible in animal models—capabilities such as isolating, tweaking, and observing the impact of specific variables in a humanized model of target physiological and pathophysiological conditions. This could substantially reduce the number of animals used in preclinical studies. Similarly, EHDA can make a marked improvement in developing targeted and on-demand DDS. However, despite its demonstrated superiority over competing methods, adoption of EHDA in these and other nanomedicine applications remains dwarfed due to its weakness in reproducibility and scale.

In the words of Melcher and Taylor regarding EHDA research, from five decades ago, “the center of attention in almost any discussion is the lack of reproducibility in experiments and the inadequacies of theoretical models… Yet the foundations of fluid mechanics are formed from work that relates carefully designed experiments to analytical models”. The note regarding lack of reproducibility and of theoretical models rings true even today. Addressing these challenges is not trivial, given the number of material, process, and environmental variables, the number and complex geometries of interfaces—which vary dramatically across facilities and applications further exacerbating matters—in EHDA assemblies, surface-active components, the complexity of interactions underlying S/IFT, and the ensuing vagaries. Ironically, the very principle—interplay among S/IFT, gravity, mechanical forces, and electric fields—bestowing upon EHDA its characteristic advantages also leaves it vulnerable to the Achilles heel of instability and unpredictability. While gravity can be assumed constant and applied voltage and mechanical force can be equipment controlled, variations in S/IFT can dramatically diminish stability, alter resulting properties, or both. Our approach for estimating S/IFT relates “carefully designed experiments” to numerical and DSP models—while making minimal changes to the assembly—to innately account for conditions specific to the EHDA site, materials, and equipment.

In comparing the du Noüy ring with their preferred Wilhelmy plate, Krüss GmbH noted that “rings are for fingers — plates are for surface tension”, which we would update to “rings are for fingers, plates are for dinner — drop kinetics are for surface tension”. Dry humor notwithstanding, as evidenced in Moore’s Law [43], nanotechnology has led to the exponential growth of *in silico* capabilities; better hardware supporting increasingly demanding software. It is thus, appropriate to complete this loop by applying advances in computational capacity, including computational modeling and DSP, toward furthering nanotechnology; specifically, nanofabrication technologies such as EHDA in developing new theranostic devices and biomimetic microphysiological systems that enhance both the quantity and quality of life of people around the world. We hope that ours is but one step toward building that feedback loop.

## Figures and Tables

**Table 1:** Summary of simulation parameters.

Simulation	Material	Density (kg/m^3^)	Viscosity (kg/m·s)	Velocity (m/s)	Time Step (s)
Water-Air	Water	998.2	0.001003	0.083	5 × 10^−6^
	Air	1.225	1.7894 × 10^−5^		
Ethanol-Air	70% Ethanol	880	0.0025	0.0415	1 × 10^−6^
	Air	1.225	1.7894 × 10^−5^		
Chloroform-Air	Chloroform	1490	0.000563	0.0415	5 × 10^−6^
	Air	1.225	1.7894 × 10^−5^		
Chloroform-Water	Chloroform	1490	0.000563	0.0415	1 × 10^−6^
	Water	998.2	0.001003		
	Air	1.225	1.7894 × 10^−5^		

## Supplementary Materials

The following are available online at https://www.mdpi.com/2072-666X/11/7/687/s1.
