# Supplementary material for: Measuring Surface and Interfacial Tension In Situ in Microdripping Mode for Electrohydrodynamic Applications
Source: Micromachines (Basel). 2020 Jul 16;11(7):687. doi: 10.3390/mi11070687 (PMC7408038; doi:10.3390/mi11070687)
Supplement: Supplementary file 1 [file micromachines-11-00687-s001.pdf]

Supplementary Materials: Measuring Surface and Interfacial Tension In Situ in Microdripping Mode for Electrohydrodynamic Applications

Karim I. Budhwani, Gerald M. Pekmezi and Mohamed M. Selim

1. Experimental Materials and Methods

Minor changes were made to setup described in earlier studies. A KDS-410 (kdScientific®, Holliston, MA) syringe-pump was connected to stainless steel 14g needle (1.600/2.108 mm inner/outer diameter. Ramé-hart co.) using 1/16″ Tygon® tubing. A Teledyne Dalsa Genie® CR-GM00-H6401 camera fitted with 10x macro lens was connected to a computer running custom LabView® 2016 (Vision Acquisition Software® 2017, and Vision Development Module® 2016) and MATLAB 2017a (MathWorks®) programs. Parameters such as nozzle diameter, flowrate, density, and viscosity were configured for a low Weber number. Droplet frequencies near 1-5 Hz minimize computational resources and control experimental variance. Conditions such as atmospheric saturation, nozzle-plane leveling, and surrounding vibration were monitored. Ethanol (70% v/v) purchased from Ricca Chemical Company (Arlington, TX). Molecular-biology-grade chloroform and non-ionic surfactant Tween® 20 purchased from Fisher Scientific (Fair Lawn, NJ). All experiments were at least performed in triplicate. At least 3 measurements were made for all experimental observations.

2. Function dkAnalyze (MATLAB® Script)

MATLAB® script encoding drop-kick algorithm for analyzing signals derived from both CFD output and CCD video.

```
function [hzDrops, sdDrops, hzWsd, wsdHarmonic, hzFft, fftHarmonic] =
dkAnalyze(peakSignal, rawSignal, fps, pathName, fileName)
% function dkAnalyze(peakSignal, rawSignal, fps, pathName, fileName)
%
% Calculate periodicity and DK frequencies for input signal "rawSignal"
% recorded at "fps" frame rate (sampling frequency) using multiple
% algorithms. Start with average drop rate from "peakSignal". Plot results
% from each algorithm on a single side-by-side figure (saved as "fileName"
% in folder "pathName") for a quick visual analytical and validation view
% of drop kinetics and periodicity.
% Spectral analysis algorithms: Welch's power spectral density and FFT.

% 1. Initialize variables
% 1a. Length of signal (# of images)
L = length(peakSignal);

% 1b. % Next power of 2 from 100 times signal length to produce output with
% more frequency bins closely spaced for higher resolution.
NFFT = 2^nextpow2(L*100);

% 1c. Handle to figure
hFigure = figure;
hFigure.Position = [100 300 1200 400];
```

```
% o o o o o o o o o o o o o o o o o o o o o o o o o o o o o o o o o o o o o

% 2. Count the drops
signalPeaks = peakSignal;                      % Use peak highlighted signal
[~, dropLocs] = findpeaks( signalPeaks );      % Get locations of those peaks
if(length(dropLocs)>14)
    dropLocs = dropLocs(2:length(dropLocs)-1);  % Skip first and last drops
end
locDiffs = sort(diff(dropLocs));               % Compute and sort drop locs
locQuartile = length(locDiffs)/4;              % Compute quartile width

% 2a. Compute periodicity of drops
if(floor(locQuartile)<1)
    hzDrops = 1/(mean(locDiffs)/fps);
else
    hzDrops = 1/(mean(locDiffs(floor(locQuartile):ceil(locQuartile*3)))/fps);
end

% 2b. Display period on screen for input into statistical analysis package
disp( [ 'Drop Kick ~DROP~', fileName, '~', num2str(1/hzDrops, '%.4f'), '~
seconds ~', num2str(hzDrops, '%.4f'), '~ Hz' ] );

% o o o o o o o o o o o o o o o o o o o o o o o o o o o o o o o o o o o o o

% 3. Plot drop kinetics
subplot(1,4,1:2);

% 3a. X-axis
t = seconds(1/fps):seconds(1/fps):seconds(L/fps);

% 3b. Compute individual drop frequencies
dropMids = diff(dropLocs)/2;
dropMids = dropMids+dropLocs(1:length(dropMids));
dropMids = seconds(dropMids/fps);
sdDrops = std(1./(diff(dropLocs)/fps));

% 3c. Plot signal with peaks highlighted to show dispensed drops
plot(t, peakSignal, '-o', 'MarkerIndices', dropLocs)
title( 'Drop Kick dS/dt' )
xlabel( 'Time (s)')
ylabel( 'd( \Sigma Intensity )' )
maxY = max(peakSignal);
yl = ylim;
ylim([yl(1), maxY*1.15]);   % Extend y-axis to accomodate sub-title
text( t(ceil(end/2)), maxY*1.1, [' \muDK: ', num2str(hzDrops, '%.4f'), ' Hz
(rsd: ', num2str(sdDrops/hzDrops, '%.3f'), ')'], 'HorizontalAlignment',
'center' );

% 3d. On secondary y-axis, plot drop-kick Hz for each drop
yyaxis right;
eachHz = 1./(diff(dropLocs)/fps);
plot(dropMids, eachHz);
yl = ylim;
ylim([yl(1)*.95, yl(2)*1.1]);
```

```
yl = ylim;
yBump = (yl(2)-yl(1))/10;
for i = 1:length(dropMids)
    h = text( dropMids(i), eachHz(i)+yBump, [num2str(eachHz(i), '%.2f'),
'Hz'], 'HorizontalAlignment', 'center', 'Color', [0.85 0.325 0.098] );
    set( h, 'Rotation', 80);
end
yticklabels([]);    % Mute labels on secondary y-axis to reduce clutter

% o o o o o o o o o o o o o o o o o o o o o o o o o o o o o o o o o o o o

% 4. Compute period from Welch's power spectral density
% Use Welch's method (P.D. Welch) to determine spectral density
% ie. power of a signal at different frequencies.
% Algorithm notes: Signal converted from time to Hz domain for
periodogram/spectrum
% Compared to standard periodogram spectrum method and Bartlett's method,
% WSD reduces noise by compromising (reducing) frequency resolution.
% Advantageous when signal contains noise from imperfect or finite data
% where noise reduction is desired.

% 4a. Get spectral density
[wsd, freq] = pwelch(rawSignal,[],[],NFFT,fps);

% 4b. Trim all frequencies above 30 Hz as they are way beoynd our range
i30Hz = find( freq < 30, 1, 'last' );
freq = freq(2:i30Hz);
wsd = wsd(2:i30Hz);

% 4c. Bracket spectrum by ignoring Hz below half the average drop rate
while( freq(1) < hzDrops*0.5 )
    freq = freq(2:length(freq)-1);
    wsd = wsd(2:length(wsd)-1);
end

% 4d. Find peak across entire spectrum
[maxAmp, iMaxAmp] = max(wsd);

% 4e. Attempt to find peak in region derived from drop rate
windowStart = find( freq < hzDrops*0.75, 1, 'last' );
if( isempty(windowStart))
    windowStart = 1;
end;
windowEnd = find( freq > hzDrops*1.25, 1 );
wsdWindow = wsd(windowStart:windowEnd);
[maxAmp, iMaxAmp] = max(wsdWindow);
iMaxAmp = iMaxAmp + windowStart - 1;

% 4f. Compute drop-kick Hz from WSD spectrum
hzWsd = freq(iMaxAmp);

% 4g. Get periodicity from first 3 peaks in WSD spectrum
wsd(~isfinite(wsd)) = 0;
wsdSmooth = smoothLowAmplitude(wsd, 0.25);
[~, wsdLocs]=findpeaks(wsdSmooth);
```

```
wsdHarmonic = median(diff(freq(wsdLocs(1:min([4 length(wsdLocs)])))));

% 4g. Plot spectrum
subplot( 1, 4, 3 );
plot( freq(1:floor(length(freq)/2)), wsd(1:floor(length(freq)/2)), 'k',
'linewidth', 1.2 );
title( 'Welch Spectral Density' )
xlabel('Frequency (Hz)')
ylabel('Spectral Density (Hz^{-1})')
yl = ylim;
maxY = min([yl(2) wsd(iMaxAmp)]);
ylim([yl(1), maxY*1.15]);
text(hzWsd, maxY*1.1, {['\leftarrow DK: ', num2str(hzWsd, '%.4f'), 'Hz'], ['
(H: ', num2str(wsdHarmonic, '%.4f'), 'Hz)']} );
hold on
plot( [hzWsd hzWsd], ylim, ':', 'color', 'black' )
hold off

disp( [ 'Drop Kick ~WSD~', fileName, '~', num2str(1/hzWsd, '%.4f'), '~
seconds ~', num2str(hzWsd, '%.4f'), '~ Hz' ] );

% o o o o o o o o o o o o o o o o o o o o o o o o o o o o o o o o o o o o

% 5. Compute period and harmonics using FFT
% Note: FFT is "conjugate symmetric" so 2-sided spectrum function of both
% positive and negative frequencies [-Fs/2 to +Fs/2).
% Compute 1-sided spectrum (with twice the amplitude) based
% on 2-sided spectrum and signal length L.
% Absolute of complex-valued FFT to get 1-sided magnitude (not phase)

fftAmp = abs(fft(rawSignal,NFFT)/L);          % |FFT Amplitude| 2-sided
fftAmp = 2*fftAmp(1:(NFFT/2)+1);              % 2*|FFT Amplitude| 1-sided
fftHz = (fps/2)*linspace(0,1,(NFFT/2)+1);     % FFT Frequency

% 5a. Trim spectrum as follows:
% Trim all frequencies above 30 Hz as they are way beoynd our range
% Ignore constant offset: fftAmp(1)
fftLen = find( fftHz < 30, 1, 'last' );
fftAmp = fftAmp(2:fftLen);
fftHz = fftHz(2:fftLen);

% 5b. Bracket spectrum by ignoring Hz below half the average drop rate
while( fftHz(1) < hzDrops*0.5 )
    fftLen = length(fftHz)-1;
    fftAmp = fftAmp(2:fftLen);
    fftHz = fftHz(2:fftLen);
end

% 5c. Find peak across entire spectrum
[spectrumPeak, iSpectrumPeak] = max(fftAmp);

% 5d. Attempt to find peak in region derived from drop rate
windowStart = find( fftHz < hzDrops*0.75, 1, 'last' );
windowEnd = find( fftHz > hzDrops*1.25, 1 );
fftWindow = fftAmp(windowStart:windowEnd);
```

```
[maxAmp, iMaxAmp] = max(fftWindow);
iMaxAmp = iMaxAmp + windowStart - 1;

% 5e. Compute drop-kick Hz from FFT spectrum
hzFft = fftHz(iMaxAmp);
hzFftDom = fftHz(iSpectrumPeak);

% 5f. Get periodicity from first 3 peaks in FFT spectrum
fftAmp(~isfinite(fftAmp)) = 0;
fftSmooth = smoothLowAmplitude(fftAmp, 0.25);
[~, fftLocs]=findpeaks(fftSmooth);
fftHarmonic = median(diff(fftHz(fftLocs(1:min([4 length(fftLocs)])))));

% 5g. Plot spectrum
subplot( 1, 4, 4 );
plot( fftHz(1:floor(length(fftHz)/2)), fftAmp(1:floor(length(fftHz)/2)) )
title( 'FFT Spectrum' )
xlabel( 'Frequency (Hz)' )
ylabel( '|Amplitude(db)|' )
yl = ylim;
maxY = min([yl(2) max(fftAmp)]);
ylim([yl(1), maxY*1.15]);
text(hzFft, maxY*1.1, {['\leftarrow DK: ', num2str(hzFft, '%.4f'), 'Hz'], ['
(H: ', num2str(fftHarmonic, '%.4f'), 'Hz)']} );
hold on
plot( [hzFft hzFft], ylim, ':', 'color', 'black' )
hold off

% 5h. Annotate if peak outside range.
if( iSpectrumPeak ~= iMaxAmp )
    disp( [ 'Drop Kick ~FFT~', fileName, '~', num2str(1/hzFft, '%.4f'), '~
seconds ~', num2str(hzFft, '%.4f'), '~ Hz. Dominant harmonic ~',
num2str(hzFftDom), '~ Hz' ] );
else
    disp( [ 'Drop Kick ~FFT~', fileName, '~', num2str(1/hzFft, '%.4f'), '~
seconds ~', num2str(hzFft, '%.4f'), '~ Hz' ] );
end

% o o o o o o o o o o o o o o o o o o o o o o o o o o o o o o o o o o o o

% 6. Save figure to PNG and EPS format files
print( hFigure, [pathName, fileName, '.Analyze.png'], '-dpng', '-r300' );
print( hFigure, [pathName, fileName, '.Analyze.eps'], '-depsc', '-r300' );

% 7. Close figure handle to clean up resources
close(hFigure);

% o o o o o o o o o o o o o o o o o o o o o o o o o o o o o o o o o o o o
```

3. Ansys® Fluent® Runtime Parameters for Water-Air SFT

Version

```
Fluent
Version: axi, pbns, vof, lam, transient (axi, pressure-based, VOF,
laminar, transient)
Release: 18.0.0
Title:
```

Models

Model	Settings
Space	Axisymmetric
Time	Unsteady, 1st-Order Implicit
Viscous	Laminar
Heat Transfer	Disabled
Solidification and Melting	Disabled
Species	Disabled
Coupled Dispersed Phase	Disabled
NOx Pollutants	Disabled
SOx Pollutants	Disabled
Soot	Disabled
Mercury Pollutants	Disabled

Material Properties

Material: water-liquid (fluid)

Property	Units	Method	Value(s)
Density	kg/m3	constant	998.2
Cp (Specific Heat)	j/kg-k	constant	4182
Thermal Conductivity	w/m-k	constant	0.6
Viscosity	kg/m-s	constant	0.001003
Molecular Weight	kg/kmol	constant	18.0152
Thermal Expansion Coefficient	1/k	constant	0
Speed of Sound	m/s	none	#f

Material: air (fluid)

Property	Units	Method	Value(s)
Density	kg/m3	constant	1.225
Cp (Specific Heat)	j/kg-k	constant	1006.43
Thermal Conductivity	w/m-k	constant	0.0242
Viscosity	kg/m-s	constant	1.7894e-05
Molecular Weight	kg/kmol	constant	28.966
Thermal Expansion Coefficient	1/k	constant	0
Speed of Sound	m/s	none	#f

Cell Zone Conditions

```
Zones

   name              id   type
   ----------------------------
   fluid_surface1    3    fluid
   filled_needle     6    fluid

Setup Conditions

   fluid_surface1

      Condition       Value
      ---------------------
      Frame Motion?   no
      Mesh Motion?    no

   filled_needle

      Condition       Value
      ---------------------
      Frame Motion?   no
      Mesh Motion?    no
```

Boundary Conditions

```
Zones

   name                             id   type
   -----------------------------------------------------
   symmetry_left_-fluid_surface1    7    symmetry
   symmetry_left_-filled_needle     8    symmetry
   symmetry_right                   9    symmetry
   outlet                           10   pressure-outlet
   inlet                            11   velocity-inlet
   wall-fluid_surface1              12   wall
   wall-filled_needle               14   wall

Setup Conditions

   symmetry_left_-fluid_surface1

      Condition   Value
      -----------------

   symmetry_left_-filled_needle

      Condition   Value
      -----------------

   symmetry_right

      Condition   Value
      -----------------

   outlet
```

Condition	Value

inlet

Condition	Value
Velocity Magnitude (m/s)	0.083

wall-fluid_surface1

Condition	Value
Wall Motion	0
Shear Boundary Condition	0
Contact Angles	(((constant . 0.78539805) (profile)))

wall-filled_needle

Condition	Value
Wall Motion	0
Shear Boundary Condition	0
Contact Angles	(((constant . 0.78539805) (profile)))

Solver Settings

Equations

Equation	Solved
Flow	yes
Volume Fraction	yes
Level-set Function	yes

Numerics

Numeric	Enabled
Absolute Velocity Formulation	yes

Unsteady Calculation Parameters

Time Step (s)	5e-06
Max. Iterations Per Time Step	10

Relaxation

Variable	Relaxation Factor
Pressure	0.3
Density	1
Body Forces	1
Momentum	0.7

Level-set Function	0.3

Linear Solver

Variable	Solver Type	Termination Criterion	Residual Reduction Tolerance
Pressure	V-Cycle	0.1	
X-Momentum	Flexible	0.1	0.7
Y-Momentum	Flexible	0.1	0.7
Level-set Function	Flexible	0.1	0.7

Pressure-Velocity Coupling

Parameter	Value
Type	SIMPLE

Discretization Scheme

Variable	Scheme
Pressure	PRESTO!
Momentum	Second Order Upwind
Volume Fraction	Geo-Reconstruct
Level-set Function	First Order Upwind

Solution Limits

Quantity	Limit
Minimum Absolute Pressure	1
Maximum Absolute Pressure	5e+10
Minimum Temperature	1
Maximum Temperature	5000

4. Ansys® Fluent® Runtime Parameters for Ethanol-Air SFT

Version

```
Fluent
Version: axi, pbns, vof, lam, transient (axi, pressure-based, VOF,
laminar, transient)
Release: 18.0.0
Title:
```

Models

```
Model                        Settings
---------------------------------------------------------
Space                        Axisymmetric
Time                         Unsteady, 1st-Order Implicit
Viscous                      Laminar
Heat Transfer                Disabled
Solidification and Melting   Disabled
Species                      Disabled
Coupled Dispersed Phase      Disabled
NOx Pollutants               Disabled
SOx Pollutants               Disabled
Soot                         Disabled
Mercury Pollutants           Disabled
```

Material Properties

```
Material: ethyl-alcohol-liquid (fluid)

   Property                        Units     Method     Value(s)
   -------------------------------------------------------------
   Density                         kg/m3     constant   880
   Cp (Specific Heat)              j/kg-k    constant   2470
   Thermal Conductivity            w/m-k     constant   0.182
   Viscosity                       kg/m-s    constant   0.0025
   Molecular Weight                kg/kmol   constant   46.07
   Thermal Expansion Coefficient   1/k       constant   0
   Speed of Sound                  m/s       none       #f

Material: air (fluid)

   Property                        Units     Method     Value(s)
   ---------------------------------------------------------------
   Density                         kg/m3     constant   1.225
   Cp (Specific Heat)              j/kg-k    constant   1006.43
   Thermal Conductivity            w/m-k     constant   0.0242
   Viscosity                       kg/m-s    constant   1.7894e-05
   Molecular Weight                kg/kmol   constant   28.966
   Thermal Expansion Coefficient   1/k       constant   0
   Speed of Sound                  m/s       none       #f
```

Cell Zone Conditions

Zones

name	id	type
fluid_surface1	3	fluid
filled_needle	6	fluid

Setup Conditions

fluid_surface1

Condition	Value
Frame Motion?	no
Mesh Motion?	no

filled_needle

Condition	Value
Frame Motion?	no
Mesh Motion?	no

Boundary Conditions

Zones

name	id	type
symmetry_left_-fluid_surface1	7	symmetry
symmetry_left_-filled_needle	8	symmetry
symmetry_right	9	symmetry
outlet	10	pressure-outlet
inlet	11	velocity-inlet
wall-fluid_surface1	12	wall
wall-filled_needle	14	wall

Setup Conditions

symmetry_left_-fluid_surface1

Condition	Value

symmetry_left_-filled_needle

Condition	Value

symmetry_right

Condition	Value

outlet

```
   Condition   Value
   -----------------

inlet

   Condition                  Value
   ---------------------------------
   Velocity Magnitude (m/s)   0.0415

wall-fluid_surface1

   Condition                  Value
   ------------------------------------------------------------------
   Wall Motion                0
   Shear Boundary Condition   0
   Contact Angles             (((constant . 0.78539805) (profile  )))

wall-filled_needle

   Condition                  Value
   ------------------------------------------------------------------
   Wall Motion                0
   Shear Boundary Condition   0
   Contact Angles             (((constant . 0.78539805) (profile  )))
```

Solver Settings

```
Equations

   Equation             Solved
   ---------------------------
   Flow                 yes
   Volume Fraction      yes
   Level-set Function   yes

Numerics

   Numeric                           Enabled
   ---------------------------------------
   Absolute Velocity Formulation     yes

Unsteady Calculation Parameters


   -------------------------------------
   Time Step (s)                     1e-06
   Max. Iterations Per Time Step     10

Relaxation

   Variable             Relaxation Factor
   --------------------------------------
   Pressure             0.3
   Density              1
   Body Forces          1
```

Momentum	0.7
Level-set Function	0.3

Linear Solver

Variable	Solver Type	Termination Criterion	Residual Reduction Tolerance
Pressure	V-Cycle	0.1	
X-Momentum	Flexible	0.1	0.7
Y-Momentum	Flexible	0.1	0.7
Level-set Function	Flexible	0.1	0.7

Pressure-Velocity Coupling

Parameter	Value
Type	SIMPLE

Discretization Scheme

Variable	Scheme
Pressure	PRESTO!
Momentum	Second Order Upwind
Volume Fraction	Geo-Reconstruct
Level-set Function	First Order Upwind

Solution Limits

Quantity	Limit
Minimum Absolute Pressure	1
Maximum Absolute Pressure	5e+10
Minimum Temperature	1
Maximum Temperature	5000

5. Ansys® Fluent® Runtime Parameters for Chloroform-Air SFT

Version

```
Fluent
Version: axi, pbns, vof, lam, transient (axi, pressure-based, VOF,
laminar, transient)
Release: 18.0.0
Title:
```

Models

```
Model                          Settings
-----------------------------------------------------------
Space                          Axisymmetric
Time                           Unsteady, 1st-Order Implicit
Viscous                        Laminar
Heat Transfer                  Disabled
Solidification and Melting     Disabled
Species                        Disabled
Coupled Dispersed Phase        Disabled
NOx Pollutants                 Disabled
SOx Pollutants                 Disabled
Soot                           Disabled
Mercury Pollutants             Disabled
```

Material Properties

```
Material: chloroform (fluid)

   Property                         Units     Method     Value(s)
   -------------------------------------------------------------
   Density                          kg/m3     constant   1490
   Cp (Specific Heat)               j/kg-k    constant   967
   Thermal Conductivity             w/m-k     constant   0.129
   Viscosity                        kg/m-s    constant   0.000563
   Molecular Weight                 kg/kmol   constant   119.3679
   Thermal Expansion Coefficient    1/k       constant   0
   Speed of Sound                   m/s       none       #f

Material: air (fluid)

   Property                         Units     Method     Value(s)
   ---------------------------------------------------------------
   Density                          kg/m3     constant   1.225
   Cp (Specific Heat)               j/kg-k    constant   1006.43
   Thermal Conductivity             w/m-k     constant   0.0242
   Viscosity                        kg/m-s    constant   1.7894e-05
   Molecular Weight                 kg/kmol   constant   28.966
   Thermal Expansion Coefficient    1/k       constant   0
   Speed of Sound                   m/s       none       #f
```

Cell Zone Conditions

Zones

name	id	type
fluid_surface1	3	fluid
filled_needle	6	fluid

Setup Conditions

fluid_surface1

Condition	Value
Frame Motion?	no
Mesh Motion?	no

filled_needle

Condition	Value
Frame Motion?	no
Mesh Motion?	no

Boundary Conditions

Zones

name	id	type
symmetry_left_-fluid_surface1	7	symmetry
symmetry_left_-filled_needle	8	symmetry
symmetry_right	9	symmetry
outlet	10	pressure-outlet
inlet	11	velocity-inlet
wall-fluid_surface1	12	wall
wall-filled_needle	14	wall

Setup Conditions

symmetry_left_-fluid_surface1

Condition	Value

symmetry_left_-filled_needle

Condition	Value

symmetry_right

Condition	Value

outlet

```
   Condition   Value
   -----------------

inlet

   Condition                  Value
   ---------------------------------
   Velocity Magnitude (m/s)   0.0415

wall-fluid_surface1

   Condition                  Value
   ------------------------------------------------------------------
   Wall Motion                0
   Shear Boundary Condition   0
   Contact Angles             (((constant . 0.78539805) (profile  )))

wall-filled_needle

   Condition                  Value
   ------------------------------------------------------------------
   Wall Motion                0
   Shear Boundary Condition   0
   Contact Angles             (((constant . 0.78539805) (profile  )))
```

Solver Settings

```
Equations

   Equation             Solved
   ---------------------------
   Flow                 yes
   Volume Fraction      yes
   Level-set Function   yes

Numerics

   Numeric                         Enabled
   ---------------------------------------
   Absolute Velocity Formulation   yes

Unsteady Calculation Parameters


   -------------------------------------
   Time Step (s)                   5e-06
   Max. Iterations Per Time Step   10

Relaxation

   Variable             Relaxation Factor
   --------------------------------------
   Pressure             0.3
   Density              1
   Body Forces          1
```

Momentum	0.7
Level-set Function	0.3

Linear Solver

Variable	Solver Type	Termination Criterion	Residual Reduction Tolerance
Pressure	V-Cycle	0.1	
X-Momentum	Flexible	0.1	0.7
Y-Momentum	Flexible	0.1	0.7
Level-set Function	Flexible	0.1	0.7

Pressure-Velocity Coupling

Parameter	Value
Type	SIMPLE

Discretization Scheme

Variable	Scheme
Pressure	PRESTO!
Momentum	Second Order Upwind
Volume Fraction	Geo-Reconstruct
Level-set Function	First Order Upwind

Solution Limits

Quantity	Limit
Minimum Absolute Pressure	1
Maximum Absolute Pressure	5e+10
Minimum Temperature	1
Maximum Temperature	5000

6. Ansys® Fluent® Runtime Parameters for Chloroform-Water IFT

Version

```
Fluent
Version: axi, pbns, vof, lam, transient (axi, pressure-based, VOF,
laminar, transient)
Release: 18.0.0
Title:
```

Models

Model	Settings
Space	Axisymmetric
Time	Unsteady, 1st-Order Implicit
Viscous	Laminar
Heat Transfer	Disabled
Solidification and Melting	Disabled
Species	Disabled
Coupled Dispersed Phase	Disabled
NOx Pollutants	Disabled
SOx Pollutants	Disabled
Soot	Disabled
Mercury Pollutants	Disabled

Material Properties

Material: water-liquid (fluid)

Property	Units	Method	Value(s)
Density	kg/m3	constant	998.2
Cp (Specific Heat)	j/kg-k	constant	4182
Thermal Conductivity	w/m-k	constant	0.6
Viscosity	kg/m-s	constant	0.001003
Molecular Weight	kg/kmol	constant	18.0152
Thermal Expansion Coefficient	1/k	constant	0
Speed of Sound	m/s	none	#f

Material: chloroform (fluid)

Property	Units	Method	Value(s)
Density	kg/m3	constant	1490
Cp (Specific Heat)	j/kg-k	constant	967
Thermal Conductivity	w/m-k	constant	0.129
Viscosity	kg/m-s	constant	0.000563
Molecular Weight	kg/kmol	constant	119.3679
Thermal Expansion Coefficient	1/k	constant	0
Speed of Sound	m/s	none	#f

Material: air (fluid)

Property	Units	Method	Value(s)
Density	kg/m3	constant	1.225
Cp (Specific Heat)	j/kg-k	constant	1006.43

Thermal Conductivity	w/m-k	constant	0.0242
Viscosity	kg/m-s	constant	1.7894e-05
Molecular Weight	kg/kmol	constant	28.966
Thermal Expansion Coefficient	1/k	constant	0
Speed of Sound	m/s	none	#f

Cell Zone Conditions

Zones

name	id	type
fluid_surface1	3	fluid
filled_needle	6	fluid

Setup Conditions

fluid_surface1

Condition	Value
Frame Motion?	no
Mesh Motion?	no

filled_needle

Condition	Value
Frame Motion?	no
Mesh Motion?	no

Boundary Conditions

Zones

name	id	type
symmetry_left_-fluid_surface1	7	symmetry
symmetry_left_-filled_needle	8	symmetry
symmetry_right	9	symmetry
outlet	10	pressure-outlet
inlet	11	velocity-inlet
wall-fluid_surface1	12	wall
wall-filled_needle	14	wall

Setup Conditions

symmetry_left_-fluid_surface1

Condition	Value

symmetry_left_-filled_needle

Condition	Value

symmetry_right

Condition	Value

outlet

Condition	Value

inlet

Condition	Value
Velocity Magnitude (m/s)	0.0415

wall-fluid_surface1

Condition	Value
Wall Motion	0
Shear Boundary Condition	0
Contact Angles	(((constant . 0.78539805) (profile)))

wall-filled_needle

Condition	Value
Wall Motion	0
Shear Boundary Condition	0
Contact Angles	(((constant . 0.78539805) (profile)))

Solver Settings

Equations

Equation	Solved
Flow	yes
Volume Fraction	yes
Level-set Function	yes

Numerics

Numeric	Enabled
Absolute Velocity Formulation	yes

Unsteady Calculation Parameters

Time Step (s)	1e-06
Max. Iterations Per Time Step	10

Relaxation

Variable	Relaxation Factor
Pressure	0.3
Density	1
Body Forces	1
Momentum	0.7
Level-set Function	0.3

Linear Solver

Variable	Solver Type	Termination Criterion	Residual Reduction Tolerance
Pressure	V-Cycle	0.1	
X-Momentum	Flexible	0.1	0.7
Y-Momentum	Flexible	0.1	0.7
Level-set Function	Flexible	0.1	0.7

Pressure-Velocity Coupling

Parameter	Value
Type	SIMPLE

Discretization Scheme

Variable	Scheme
Pressure	PRESTO!
Momentum	Second Order Upwind
Volume Fraction	Geo-Reconstruct
Level-set Function	First Order Upwind

Solution Limits

Quantity	Limit
Minimum Absolute Pressure	1
Maximum Absolute Pressure	5e+10
Minimum Temperature	1
Maximum Temperature	5000

7. List of LabVIEW® SubVIs and Express VIs

Low-Level VI (Virtual Instrument) to acquire "drop-kicks" video which is essentially a set of images captures at the specified frame rate from connected CCD camera.

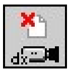 NI_Vision_Acquisition_Software.lvlib:IMAQdx Close Camera.vi

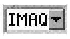 IMAQdx.ctl

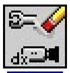 NI_Vision_Acquisition_Software.lvlib:IMAQdx Unconfigure Acquisition.vi

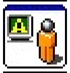 subDisplayMessage.vi

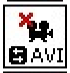 IMAQ AVI Close

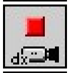 NI_Vision_Acquisition_Software.lvlib:IMAQdx Stop Acquisition.vi

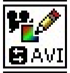 IMAQ AVI Write Frame

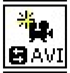 IMAQ AVI Create

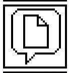 subFile Dialog.vi

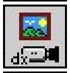 NI_Vision_Acquisition_Software.lvlib:IMAQdx Get Image.vi

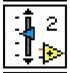 NI_Vision_Acquisition_Software.lvlib:IMAQdx Buffer Number Mode.ctl

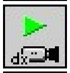 NI_Vision_Acquisition_Software.lvlib:IMAQdx Start Acquisition.vi

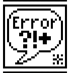 General Error Handler.vi

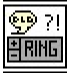 DialogType.ctl

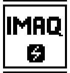 IMAQ Create

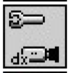 NI_Vision_Acquisition_Software.lvlib:IMAQdx Configure Acquisition.vi

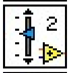 NI_Vision_Acquisition_Software.lvlib:IMAQdx Acquisition Mode.ctl

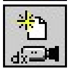 NI_Vision_Acquisition_Software.lvlib:IMAQdx Open Camera.vi

VI Revision History
"DalsaControl3.0.DropKicks.vi History"
Current Revision: 216

8. Frequencies for SFT/IFT from CFD Solution

Material	Q (mL/min)	γ (dyne/cm)	hzDrop	hzWsd	hzFft	hzMean	hzSd
70etOH-Air	5	24	5.4289	5.3978	5.3978	5.4082	0.3%
70etOH-Air	5	25	5.1020	5.0888	5.0850	5.0919	0.2%
70etOH-Air	5	26	5.0505	5.0392	5.0392	5.0430	0.1%
70etOH-Air	5	27	4.8077	4.8027	4.7989	4.8031	0.1%
70etOH-Air	5	28	4.6339	4.6158	4.6082	4.6193	0.3%
70etOH-Air	5	29	4.4297	4.4441	4.4174	4.4304	0.3%
70etOH-Air	5	30	4.4170	4.4136	4.4136	4.4147	0.0%
Water-Air	5	40	3.7783	3.7479	3.7479	3.7581	0.5%
Water-Air	5	42	3.6810	3.6564	3.6697	3.6690	0.3%
Water-Air	5	45	3.4412	3.4161	3.4180	3.4251	0.4%
Water-Air	5	47	3.3311	3.3131	3.3131	3.3191	0.3%
Water-Air	5	50	3.1070	3.1223	3.1109	3.1134	0.3%
Water-Air	5	52	3.0659	3.0212	3.0136	3.0336	0.9%
Water-Air	5	55	2.9795	2.9411	2.9354	2.9520	0.8%
Water-Air	5	57	2.8289	2.7885	2.7714	2.7963	1.1%
Water-Air	5	62	2.6178	2.6112	2.5806	2.6032	0.8%
Water-Air	5	65	2.5641	2.5387	2.5311	2.5446	0.7%
Water-Air	5	67	2.5291	2.4910	2.5139	2.5113	0.8%
Water-Air	5	70	2.4450	2.4338	2.4319	2.4369	0.3%
Water-Air	5	71.5	2.3627	2.3441	2.3270	2.3446	0.8%
Water-Air	5	72	2.2379	2.2087	2.2163	2.2210	0.7%
Water-Air	5	72.5	2.2379	2.2087	2.2163	2.2210	0.7%
Water-Air	5	75	2.1708	2.2106	2.1362	2.1725	1.7%
ChCl3-Air	3	24	5.4306	5.4302	5.4264	5.4291	0.0%
ChCl3-Air	3	25	5.2711	5.2605	5.2567	5.2627	0.1%
ChCl3-Air	3	26	5.1238	5.1041	5.1041	5.1107	0.2%
ChCl3-Air	3	27	4.8980	4.8828	4.8828	4.8879	0.2%
ChCl3-Air	3	28	4.7244	4.7188	4.7188	4.7207	0.1%
ChCl3-Air	3	29	4.6125	4.5891	4.5891	4.5969	0.3%
ChCl3-Water	5	28	2.4651	2.3842	2.4643	2.4379	1.9%
ChCl3-Water	5	29	2.3943	2.3270	2.3880	2.3697	1.6%
ChCl3-Water	5	30	2.3274	2.2087	2.3270	2.2877	3.0%
ChCl3-Water	5	31	2.2624	2.1362	2.2659	2.2215	3.3%
ChCl3-Water	5	32	2.2010	2.0561	2.2011	2.1527	3.9%
ChCl3-Water	5	33	2.1398	2.0256	2.1400	2.1018	3.1%

9. Interpolated Adjusted CFD Frequencies for Water-Air SFT @ 5 mL/minute

γ (dyne/cm)	hzAdj	γ (dyne/cm)	hzAdj	γ (dyne/cm)	hzAdj	γ (dyne/cm)	hzAdj
**40.00**	3.5980	**41.90**	3.5279	**43.80**	3.3783	**45.70**	3.2438
**40.05**	3.5970	**41.95**	3.5254	**43.85**	3.3737	**45.75**	3.2414
**40.10**	3.5959	**42.00**	3.5229	**43.90**	3.3691	**45.80**	3.2390
**40.15**	3.5947	**42.05**	3.5204	**43.95**	3.3645	**45.85**	3.2367
**40.20**	3.5935	**42.10**	3.5177	**44.00**	3.3599	**45.90**	3.2343
**40.25**	3.5923	**42.15**	3.5149	**44.05**	3.3554	**45.95**	3.2320
**40.30**	3.5910	**42.20**	3.5119	**44.10**	3.3509	**46.00**	3.2297
**40.35**	3.5896	**42.25**	3.5089	**44.15**	3.3465	**46.05**	3.2274
**40.40**	3.5882	**42.30**	3.5057	**44.20**	3.3421	**46.10**	3.2251
**40.45**	3.5868	**42.35**	3.5024	**44.25**	3.3377	**46.15**	3.2228
**40.50**	3.5853	**42.40**	3.4990	**44.30**	3.3334	**46.20**	3.2204
**40.55**	3.5837	**42.45**	3.4955	**44.35**	3.3292	**46.25**	3.2181
**40.60**	3.5821	**42.50**	3.4919	**44.40**	3.3250	**46.30**	3.2158
**40.65**	3.5805	**42.55**	3.4882	**44.45**	3.3209	**46.35**	3.2135
**40.70**	3.5788	**42.60**	3.4845	**44.50**	3.3169	**46.40**	3.2111
**40.75**	3.5771	**42.65**	3.4806	**44.55**	3.3129	**46.45**	3.2087
**40.80**	3.5753	**42.70**	3.4767	**44.60**	3.3090	**46.50**	3.2064
**40.85**	3.5735	**42.75**	3.4727	**44.65**	3.3052	**46.55**	3.2039
**40.90**	3.5717	**42.80**	3.4686	**44.70**	3.3015	**46.60**	3.2015
**40.95**	3.5698	**42.85**	3.4644	**44.75**	3.2979	**46.65**	3.1990
**41.00**	3.5679	**42.90**	3.4602	**44.80**	3.2943	**46.70**	3.1965
**41.05**	3.5659	**42.95**	3.4560	**44.85**	3.2909	**46.75**	3.1940
**41.10**	3.5640	**43.00**	3.4516	**44.90**	3.2876	**46.80**	3.1914
**41.15**	3.5619	**43.05**	3.4472	**44.95**	3.2844	**46.85**	3.1887
**41.20**	3.5599	**43.10**	3.4428	**45.00**	3.2813	**46.90**	3.1861
**41.25**	3.5578	**43.15**	3.4384	**45.05**	3.2782	**46.95**	3.1833
**41.30**	3.5557	**43.20**	3.4338	**45.10**	3.2753	**47.00**	3.1805
**41.35**	3.5535	**43.25**	3.4293	**45.15**	3.2724	**47.05**	3.1777
**41.40**	3.5513	**43.30**	3.4247	**45.20**	3.2696	**47.10**	3.1748
**41.45**	3.5491	**43.35**	3.4201	**45.25**	3.2668	**47.15**	3.1719
**41.50**	3.5468	**43.40**	3.4155	**45.30**	3.2641	**47.20**	3.1690
**41.55**	3.5446	**43.45**	3.4109	**45.35**	3.2614	**47.25**	3.1659
**41.60**	3.5423	**43.50**	3.4062	**45.40**	3.2587	**47.30**	3.1629
**41.65**	3.5399	**43.55**	3.4016	**45.45**	3.2562	**47.35**	3.1598
**41.70**	3.5376	**43.60**	3.3969	**45.50**	3.2536	**47.40**	3.1567
**41.75**	3.5352	**43.65**	3.3923	**45.55**	3.2511	**47.45**	3.1536
**41.80**	3.5328	**43.70**	3.3876	**45.60**	3.2486	**47.50**	3.1504
**41.85**	3.5304	**43.75**	3.3829	**45.65**	3.2462	**47.55**	3.1472

γ (dyne/cm)	hzAdj	γ (dyne/cm)	hzAdj	γ (dyne/cm)	hzAdj	γ (dyne/cm)	hzAdj
**47.60**	3.1439	**49.55**	3.0125	**51.50**	2.9120	**53.45**	2.8588
**47.65**	3.1407	**49.60**	3.0094	**51.55**	2.9099	**53.50**	2.8578
**47.70**	3.1374	**49.65**	3.0064	**51.60**	2.9079	**53.55**	2.8568
**47.75**	3.1341	**49.70**	3.0034	**51.65**	2.9059	**53.60**	2.8559
**47.80**	3.1307	**49.75**	3.0005	**51.70**	2.9039	**53.65**	2.8549
**47.85**	3.1274	**49.80**	2.9976	**51.75**	2.9020	**53.70**	2.8539
**47.90**	3.1240	**49.85**	2.9947	**51.80**	2.9001	**53.75**	2.8529
**47.95**	3.1206	**49.90**	2.9919	**51.85**	2.8983	**53.80**	2.8518
**48.00**	3.1172	**49.95**	2.9892	**51.90**	2.8965	**53.85**	2.8508
**48.05**	3.1138	**50.00**	2.9865	**51.95**	2.8948	**53.90**	2.8497
**48.10**	3.1103	**50.05**	2.9838	**52.00**	2.8931	**53.95**	2.8487
**48.15**	3.1069	**50.10**	2.9811	**52.05**	2.8914	**54.00**	2.8475
**48.20**	3.1034	**50.15**	2.9784	**52.10**	2.8898	**54.05**	2.8464
**48.25**	3.0999	**50.20**	2.9757	**52.15**	2.8883	**54.10**	2.8453
**48.30**	3.0965	**50.25**	2.9731	**52.20**	2.8868	**54.15**	2.8441
**48.35**	3.0930	**50.30**	2.9704	**52.25**	2.8854	**54.20**	2.8429
**48.40**	3.0895	**50.35**	2.9678	**52.30**	2.8839	**54.25**	2.8416
**48.45**	3.0860	**50.40**	2.9651	**52.35**	2.8826	**54.30**	2.8404
**48.50**	3.0826	**50.45**	2.9625	**52.40**	2.8812	**54.35**	2.8391
**48.55**	3.0791	**50.50**	2.9599	**52.45**	2.8800	**54.40**	2.8377
**48.60**	3.0756	**50.55**	2.9573	**52.50**	2.8787	**54.45**	2.8363
**48.65**	3.0721	**50.60**	2.9547	**52.55**	2.8775	**54.50**	2.8349
**48.70**	3.0687	**50.65**	2.9521	**52.60**	2.8763	**54.55**	2.8334
**48.75**	3.0652	**50.70**	2.9496	**52.65**	2.8751	**54.60**	2.8319
**48.80**	3.0618	**50.75**	2.9470	**52.70**	2.8740	**54.65**	2.8304
**48.85**	3.0583	**50.80**	2.9445	**52.75**	2.8728	**54.70**	2.8288
**48.90**	3.0549	**50.85**	2.9420	**52.80**	2.8717	**54.75**	2.8271
**48.95**	3.0515	**50.90**	2.9395	**52.85**	2.8707	**54.80**	2.8254
**49.00**	3.0481	**50.95**	2.9371	**52.90**	2.8696	**54.85**	2.8236
**49.05**	3.0448	**51.00**	2.9347	**52.95**	2.8686	**54.90**	2.8218
**49.10**	3.0414	**51.05**	2.9323	**53.00**	2.8676	**54.95**	2.8199
**49.15**	3.0381	**51.10**	2.9299	**53.05**	2.8666	**55.00**	2.8180
**49.20**	3.0348	**51.15**	2.9275	**53.10**	2.8656	**55.05**	2.8159
**49.25**	3.0315	**51.20**	2.9252	**53.15**	2.8646	**55.10**	2.8136
**49.30**	3.0283	**51.25**	2.9229	**53.20**	2.8636	**55.15**	2.8110
**49.35**	3.0250	**51.30**	2.9207	**53.25**	2.8626	**55.20**	2.8082
**49.40**	3.0218	**51.35**	2.9184	**53.30**	2.8617	**55.25**	2.8051
**49.45**	3.0187	**51.40**	2.9163	**53.35**	2.8607	**55.30**	2.8019
**49.50**	3.0156	**51.45**	2.9141	**53.40**	2.8597	**55.35**	2.7985

γ (dyne/cm)	hzAdj	γ (dyne/cm)	hzAdj	γ (dyne/cm)	hzAdj	γ (dyne/cm)	hzAdj
**55.40**	2.7949	**57.35**	2.6425	**59.30**	2.5568	**61.25**	2.4951
**55.45**	2.7912	**57.40**	2.6400	**59.35**	2.5549	**61.30**	2.4938
**55.50**	2.7873	**57.45**	2.6375	**59.40**	2.5531	**61.35**	2.4926
**55.55**	2.7832	**57.50**	2.6350	**59.45**	2.5512	**61.40**	2.4913
**55.60**	2.7791	**57.55**	2.6325	**59.50**	2.5494	**61.45**	2.4901
**55.65**	2.7748	**57.60**	2.6301	**59.55**	2.5476	**61.50**	2.4889
**55.70**	2.7704	**57.65**	2.6277	**59.60**	2.5458	**61.55**	2.4876
**55.75**	2.7659	**57.70**	2.6253	**59.65**	2.5440	**61.60**	2.4865
**55.80**	2.7614	**57.75**	2.6229	**59.70**	2.5423	**61.65**	2.4853
**55.85**	2.7567	**57.80**	2.6205	**59.75**	2.5405	**61.70**	2.4841
**55.90**	2.7521	**57.85**	2.6181	**59.80**	2.5388	**61.75**	2.4830
**55.95**	2.7474	**57.90**	2.6158	**59.85**	2.5371	**61.80**	2.4818
**56.00**	2.7426	**57.95**	2.6135	**59.90**	2.5353	**61.85**	2.4807
**56.05**	2.7378	**58.00**	2.6112	**59.95**	2.5337	**61.90**	2.4796
**56.10**	2.7331	**58.05**	2.6089	**60.00**	2.5320	**61.95**	2.4785
**56.15**	2.7283	**58.10**	2.6066	**60.05**	2.5303	**62.00**	2.4774
**56.20**	2.7236	**58.15**	2.6043	**60.10**	2.5287	**62.05**	2.4764
**56.25**	2.7189	**58.20**	2.6021	**60.15**	2.5271	**62.10**	2.4753
**56.30**	2.7142	**58.25**	2.5999	**60.20**	2.5255	**62.15**	2.4742
**56.35**	2.7096	**58.30**	2.5976	**60.25**	2.5239	**62.20**	2.4732
**56.40**	2.7051	**58.35**	2.5954	**60.30**	2.5223	**62.25**	2.4722
**56.45**	2.7006	**58.40**	2.5933	**60.35**	2.5207	**62.30**	2.4712
**56.50**	2.6963	**58.45**	2.5911	**60.40**	2.5192	**62.35**	2.4702
**56.55**	2.6920	**58.50**	2.5890	**60.45**	2.5176	**62.40**	2.4692
**56.60**	2.6879	**58.55**	2.5868	**60.50**	2.5161	**62.45**	2.4682
**56.65**	2.6839	**58.60**	2.5847	**60.55**	2.5146	**62.50**	2.4672
**56.70**	2.6800	**58.65**	2.5826	**60.60**	2.5131	**62.55**	2.4662
**56.75**	2.6763	**58.70**	2.5805	**60.65**	2.5116	**62.60**	2.4653
**56.80**	2.6728	**58.75**	2.5785	**60.70**	2.5102	**62.65**	2.4643
**56.85**	2.6694	**58.80**	2.5764	**60.75**	2.5087	**62.70**	2.4634
**56.90**	2.6662	**58.85**	2.5744	**60.80**	2.5073	**62.75**	2.4624
**56.95**	2.6633	**58.90**	2.5724	**60.85**	2.5059	**62.80**	2.4615
**57.00**	2.6605	**58.95**	2.5704	**60.90**	2.5045	**62.85**	2.4606
**57.05**	2.6579	**59.00**	2.5684	**60.95**	2.5031	**62.90**	2.4597
**57.10**	2.6553	**59.05**	2.5664	**61.00**	2.5017	**62.95**	2.4588
**57.15**	2.6527	**59.10**	2.5645	**61.05**	2.5004	**63.00**	2.4579
**57.20**	2.6501	**59.15**	2.5625	**61.10**	2.4990	**63.05**	2.4570
**57.25**	2.6475	**59.20**	2.5606	**61.15**	2.4977	**63.10**	2.4562
**57.30**	2.6450	**59.25**	2.5587	**61.20**	2.4964	**63.15**	2.4553

γ (dyne/cm)	hzAdj
**63.20**	2.4545
**63.25**	2.4536
**63.30**	2.4528
**63.35**	2.4520
**63.40**	2.4512
**63.45**	2.4504
**63.50**	2.4496
**63.55**	2.4488
**63.60**	2.4480
**63.65**	2.4472
**63.70**	2.4465
**63.75**	2.4457
**63.80**	2.4450
**63.85**	2.4443
**63.90**	2.4435
**63.95**	2.4428
**64.00**	2.4421
**64.05**	2.4414
**64.10**	2.4407
**64.15**	2.4400
**64.20**	2.4394
**64.25**	2.4387
**64.30**	2.4381
**64.35**	2.4374
**64.40**	2.4368
**64.45**	2.4362
**64.50**	2.4355
**64.55**	2.4349
**64.60**	2.4343
**64.65**	2.4337
**64.70**	2.4331
**64.75**	2.4326
**64.80**	2.4320
**64.85**	2.4314
**64.90**	2.4309
**64.95**	2.4303
**65.00**	2.4298
**65.05**	2.4293
**65.10**	2.4288

γ (dyne/cm)	hzAdj
**65.15**	2.4283
**65.20**	2.4278
**65.25**	2.4274
**65.30**	2.4270
**65.35**	2.4266
**65.40**	2.4262
**65.45**	2.4258
**65.50**	2.4254
**65.55**	2.4250
**65.60**	2.4247
**65.65**	2.4243
**65.70**	2.4240
**65.75**	2.4236
**65.80**	2.4233
**65.85**	2.4229
**65.90**	2.4226
**65.95**	2.4223
**66.00**	2.4219
**66.05**	2.4216
**66.10**	2.4213
**66.15**	2.4209
**66.20**	2.4206
**66.25**	2.4202
**66.30**	2.4199
**66.35**	2.4195
**66.40**	2.4191
**66.45**	2.4187
**66.50**	2.4183
**66.55**	2.4179
**66.60**	2.4175
**66.65**	2.4170
**66.70**	2.4166
**66.75**	2.4161
**66.80**	2.4156
**66.85**	2.4150
**66.90**	2.4145
**66.95**	2.4139
**67.00**	2.4133
**67.05**	2.4127

γ (dyne/cm)	hzAdj
**67.10**	2.4121
**67.15**	2.4114
**67.20**	2.4107
**67.25**	2.4100
**67.30**	2.4093
**67.35**	2.4085
**67.40**	2.4077
**67.45**	2.4069
**67.50**	2.4060
**67.55**	2.4052
**67.60**	2.4043
**67.65**	2.4034
**67.70**	2.4025
**67.75**	2.4015
**67.80**	2.4005
**67.85**	2.3995
**67.90**	2.3985
**67.95**	2.3974
**68.00**	2.3963
**68.05**	2.3952
**68.10**	2.3941
**68.15**	2.3930
**68.20**	2.3918
**68.25**	2.3906
**68.30**	2.3894
**68.35**	2.3882
**68.40**	2.3869
**68.45**	2.3856
**68.50**	2.3843
**68.55**	2.3830
**68.60**	2.3816
**68.65**	2.3802
**68.70**	2.3788
**68.75**	2.3774
**68.80**	2.3760
**68.85**	2.3745
**68.90**	2.3730
**68.95**	2.3715
**69.00**	2.3699

γ (dyne/cm)	hzAdj
**69.05**	2.3684
**69.10**	2.3668
**69.15**	2.3652
**69.20**	2.3636
**69.25**	2.3619
**69.30**	2.3603
**69.35**	2.3586
**69.40**	2.3569
**69.45**	2.3551
**69.50**	2.3534
**69.55**	2.3516
**69.60**	2.3498
**69.65**	2.3480
**69.70**	2.3461
**69.75**	2.3442
**69.80**	2.3424
**69.85**	2.3404
**69.90**	2.3385
**69.95**	2.3366
**70.00**	2.3346
**70.05**	2.3325
**70.10**	2.3303
**70.15**	2.3279
**70.20**	2.3253
**70.25**	2.3226
**70.30**	2.3197
**70.35**	2.3167
**70.40**	2.3136
**70.45**	2.3104
**70.50**	2.3071
**70.55**	2.3037
**70.60**	2.3002
**70.65**	2.2966
**70.70**	2.2930
**70.75**	2.2893
**70.80**	2.2856
**70.85**	2.2818
**70.90**	2.2780
**70.95**	2.2742

γ (dyne/cm)	hzAdj
**71.00**	2.2704
**71.05**	2.2666
**71.10**	2.2628
**71.15**	2.2590
**71.20**	2.2553
**71.25**	2.2516
**71.30**	2.2479
**71.35**	2.2443
**71.40**	2.2407
**71.45**	2.2373
**71.50**	2.2339
**71.55**	2.2307
**71.60**	2.2278
**71.65**	2.2251
**71.70**	2.2226
**71.75**	2.2202
**71.80**	2.2178
**71.85**	2.2153
**71.90**	2.2127
**71.95**	2.2100
**72.00**	2.2070

γ (dyne/cm)	hzAdj
**72.05**	2.2038
**72.10**	2.2002
**72.15**	2.1961
**72.20**	2.1916
**72.25**	2.1865
**72.30**	2.1808
**72.35**	2.1708
**72.40**	2.1556
**72.45**	2.1398
**72.50**	2.1277
**72.55**	2.1190
**72.60**	2.1107
**72.65**	2.1033
**72.70**	2.0970
**72.75**	2.0922
**72.80**	2.0892
**72.85**	2.0874
**72.90**	2.0855
**72.95**	2.0838
**73.00**	2.0820
**73.05**	2.0804

γ (dyne/cm)	hzAdj
**73.10**	2.0788
**73.15**	2.0772
**73.20**	2.0757
**73.25**	2.0742
**73.30**	2.0728
**73.35**	2.0714
**73.40**	2.0701
**73.45**	2.0689
**73.50**	2.0676
**73.55**	2.0665
**73.60**	2.0653
**73.65**	2.0643
**73.70**	2.0632
**73.75**	2.0622
**73.80**	2.0613
**73.85**	2.0604
**73.90**	2.0595
**73.95**	2.0587
**74.00**	2.0579
**74.05**	2.0572
**74.10**	2.0565

γ (dyne/cm)	hzAdj
**74.15**	2.0559
**74.20**	2.0553
**74.25**	2.0547
**74.30**	2.0542
**74.35**	2.0537
**74.40**	2.0533
**74.45**	2.0529
**74.50**	2.0525
**74.55**	2.0522
**74.60**	2.0519
**74.65**	2.0516
**74.70**	2.0514
**74.75**	2.0512
**74.80**	2.0510
**74.85**	2.0509
**74.90**	2.0508
**74.95**	2.0508
**75.00**	2.0508

10. Interpolated Adjusted CFD Frequencies for 70% Ethanol-Air SFT @ 5 mL/minute

γ (dyne/cm)	hzAdj
**24.00**	5.4788
**24.05**	5.4562
**24.10**	5.4339
**24.15**	5.4119
**24.20**	5.3904
**24.25**	5.3692
**24.30**	5.3487
**24.35**	5.3288
**24.40**	5.3096
**24.45**	5.2911
**24.50**	5.2735
**24.55**	5.2569
**24.60**	5.2412
**24.65**	5.2266
**24.70**	5.2132
**24.75**	5.2010
**24.80**	5.1902
**24.85**	5.1807
**24.90**	5.1726
**24.95**	5.1662
**25.00**	5.1613
**25.05**	5.1575
**25.10**	5.1541
**25.15**	5.1512
**25.20**	5.1486
**25.25**	5.1463
**25.30**	5.1442
**25.35**	5.1424
**25.40**	5.1407
**25.45**	5.1392
**25.50**	5.1377

γ (dyne/cm)	hzAdj
**25.55**	5.1362
**25.60**	5.1346
**25.65**	5.1330
**25.70**	5.1312
**25.75**	5.1292
**25.80**	5.1270
**25.85**	5.1245
**25.90**	5.1217
**25.95**	5.1185
**26.00**	5.1148
**26.05**	5.1100
**26.10**	5.1036
**26.15**	5.0957
**26.20**	5.0864
**26.25**	5.0758
**26.30**	5.0642
**26.35**	5.0517
**26.40**	5.0384
**26.45**	5.0245
**26.50**	5.0101
**26.55**	4.9953
**26.60**	4.9803
**26.65**	4.9653
**26.70**	4.9504
**26.75**	4.9357
**26.80**	4.9214
**26.85**	4.9076
**26.90**	4.8945
**26.95**	4.8822
**27.00**	4.8709
**27.05**	4.8602

γ (dyne/cm)	hzAdj
**27.10**	4.8497
**27.15**	4.8394
**27.20**	4.8293
**27.25**	4.8193
**27.30**	4.8095
**27.35**	4.7998
**27.40**	4.7902
**27.45**	4.7807
**27.50**	4.7713
**27.55**	4.7619
**27.60**	4.7526
**27.65**	4.7433
**27.70**	4.7340
**27.75**	4.7246
**27.80**	4.7153
**27.85**	4.7059
**27.90**	4.6964
**27.95**	4.6869
**28.00**	4.6773
**28.05**	4.6672
**28.10**	4.6562
**28.15**	4.6447
**28.20**	4.6326
**28.25**	4.6202
**28.30**	4.6075
**28.35**	4.5947
**28.40**	4.5820
**28.45**	4.5694
**28.50**	4.5572
**28.55**	4.5455
**28.60**	4.5343

γ (dyne/cm)	hzAdj
**28.65**	4.5239
**28.70**	4.5144
**28.75**	4.5059
**28.80**	4.4986
**28.85**	4.4926
**28.90**	4.4880
**28.95**	4.4850
**29.00**	4.4837
**29.05**	4.4833
**29.10**	4.4830
**29.15**	4.4826
**29.20**	4.4823
**29.25**	4.4820
**29.30**	4.4817
**29.35**	4.4815
**29.40**	4.4812
**29.45**	4.4810
**29.50**	4.4808
**29.55**	4.4806
**29.60**	4.4804
**29.65**	4.4803
**29.70**	4.4802
**29.75**	4.4801
**29.80**	4.4800
**29.85**	4.4799
**29.90**	4.4798
**29.95**	4.4798
**30.00**	4.4798

11. Interpolated Adjusted CFD Frequencies for Chloroform-Air SFT @ 3 mL/minute

γ (dyne/cm)	hzAdj	γ (dyne/cm)	hzAdj	γ (dyne/cm)	hzAdj	γ (dyne/cm)	hzAdj
**24.00**	5.5078	**25.30**	5.2896	**26.60**	5.0450	**27.90**	4.8044
**24.05**	5.4988	**25.35**	5.2823	**26.65**	5.0330	**27.95**	4.7969
**24.10**	5.4898	**25.40**	5.2751	**26.70**	5.0211	**28.00**	4.7896
**24.15**	5.4808	**25.45**	5.2679	**26.75**	5.0094	**28.05**	4.7822
**24.20**	5.4719	**25.50**	5.2606	**26.80**	4.9980	**28.10**	4.7750
**24.25**	5.4631	**25.55**	5.2533	**26.85**	4.9869	**28.15**	4.7678
**24.30**	5.4543	**25.60**	5.2459	**26.90**	4.9762	**28.20**	4.7607
**24.35**	5.4455	**25.65**	5.2384	**26.95**	4.9659	**28.25**	4.7537
**24.40**	5.4367	**25.70**	5.2308	**27.00**	4.9561	**28.30**	4.7467
**24.45**	5.4281	**25.75**	5.2230	**27.05**	4.9466	**28.35**	4.7398
**24.50**	5.4194	**25.80**	5.2150	**27.10**	4.9372	**28.40**	4.7330
**24.55**	5.4108	**25.85**	5.2068	**27.15**	4.9280	**28.45**	4.7263
**24.60**	5.4023	**25.90**	5.1983	**27.20**	4.9190	**28.50**	4.7196
**24.65**	5.3938	**25.95**	5.1896	**27.25**	4.9100	**28.55**	4.7131
**24.70**	5.3853	**26.00**	5.1806	**27.30**	4.9012	**28.60**	4.7066
**24.75**	5.3769	**26.05**	5.1712	**27.35**	4.8926	**28.65**	4.7002
**24.80**	5.3685	**26.10**	5.1612	**27.40**	4.8840	**28.70**	4.6939
**24.85**	5.3602	**26.15**	5.1508	**27.45**	4.8756	**28.75**	4.6877
**24.90**	5.3519	**26.20**	5.1399	**27.50**	4.8673	**28.80**	4.6815
**24.95**	5.3437	**26.25**	5.1287	**27.55**	4.8591	**28.85**	4.6755
**25.00**	5.3355	**26.30**	5.1172	**27.60**	4.8510	**28.90**	4.6695
**25.05**	5.3275	**26.35**	5.1055	**27.65**	4.8430	**28.95**	4.6637
**25.10**	5.3196	**26.40**	5.0935	**27.70**	4.8351	**29.00**	4.6579
**25.15**	5.3119	**26.45**	5.0815	**27.75**	4.8273		
**25.20**	5.3044	**26.50**	5.0693	**27.80**	4.8196		
**25.25**	5.2970	**26.55**	5.0572	**27.85**	4.8120		

12. Interpolated Adjusted CFD Frequencies for Chloroform-Water IFT @ 5 mL/minute

γ (dyne/cm)	hzAdj
**28.00**	2.5013
**28.05**	2.4970
**28.10**	2.4928
**28.15**	2.4886
**28.20**	2.4845
**28.25**	2.4804
**28.30**	2.4763
**28.35**	2.4723
**28.40**	2.4683
**28.45**	2.4644
**28.50**	2.4605
**28.55**	2.4566
**28.60**	2.4528
**28.65**	2.4490
**28.70**	2.4453
**28.75**	2.4416
**28.80**	2.4380
**28.85**	2.4344
**28.90**	2.4308
**28.95**	2.4273
**29.00**	2.4238
**29.05**	2.4204
**29.10**	2.4171
**29.15**	2.4138
**29.20**	2.4105
**29.25**	2.4074

γ (dyne/cm)	hzAdj
**29.30**	2.4042
**29.35**	2.4011
**29.40**	2.3980
**29.45**	2.3950
**29.50**	2.3920
**29.55**	2.3890
**29.60**	2.3860
**29.65**	2.3830
**29.70**	2.3800
**29.75**	2.3770
**29.80**	2.3740
**29.85**	2.3710
**29.90**	2.3680
**29.95**	2.3650
**30.00**	2.3619
**30.05**	2.3588
**30.10**	2.3557
**30.15**	2.3526
**30.20**	2.3495
**30.25**	2.3465
**30.30**	2.3434
**30.35**	2.3403
**30.40**	2.3373
**30.45**	2.3342
**30.50**	2.3311
**30.55**	2.3281

γ (dyne/cm)	hzAdj
**30.60**	2.3250
**30.65**	2.3219
**30.70**	2.3188
**30.75**	2.3157
**30.80**	2.3125
**30.85**	2.3094
**30.90**	2.3063
**30.95**	2.3031
**31.00**	2.2999
**31.05**	2.2967
**31.10**	2.2935
**31.15**	2.2902
**31.20**	2.2869
**31.25**	2.2837
**31.30**	2.2803
**31.35**	2.2770
**31.40**	2.2737
**31.45**	2.2703
**31.50**	2.2670
**31.55**	2.2637
**31.60**	2.2603
**31.65**	2.2570
**31.70**	2.2537
**31.75**	2.2504
**31.80**	2.2471
**31.85**	2.2438

γ (dyne/cm)	hzAdj
**31.90**	2.2405
**31.95**	2.2373
**32.00**	2.2341
**32.05**	2.2309
**32.10**	2.2277
**32.15**	2.2246
**32.20**	2.2214
**32.25**	2.2183
**32.30**	2.2151
**32.35**	2.2120
**32.40**	2.2089
**32.45**	2.2057
**32.50**	2.2026
**32.55**	2.1996
**32.60**	2.1965
**32.65**	2.1934
**32.70**	2.1903
**32.75**	2.1873
**32.80**	2.1842
**32.85**	2.1812
**32.90**	2.1782
**32.95**	2.1752
**33.00**	2.1721
